# Long-Term High-Flow Nasal Therapy in Patients with Bronchiectasis of Different Severity: A Retrospective Cohort Study

**DOI:** 10.3390/jcm13206146

**Published:** 2024-10-15

**Authors:** Cecilia Calabrese, Santi Nolasco, Anna Annunziata, Alessio Sola, Pasquale Imitazione, Raffaele Campisi, Francesca Simioli, Marco Balestrino, Laura Ferrentino, Carlo Vancheri, Claudia Crimi, Giuseppe Fiorentino

**Affiliations:** 1 Department of Translational Medical Sciences, University of Campania “Luigi Vanvitelli”, Azienda Ospedaliera di Rilievo Nazionale dei Colli, Monaldi Hospital, 80131 Naples, Italy; alessio.sola.26.1224@gmail.com (A.S.); marcobalestrino.mb@gmail.com (M.B.); 2 Department of Clinical and Experimental Medicine, University of Catania, 95124 Catania, Italy; nolascos@hotmail.it (S.N.); vancheri@unict.it (C.V.); dott.claudiacrimi@gmail.com (C.C.); 3 Respiratory Medicine Unit, Policlinico “G. Rodolico-San Marco” University Hospital, 95123 Catania, Italy; raffaelemd@hotmail.it; 4 Department of Respiratory Pathophysiology and Rehabilitation, Monaldi Hospital, 80131 Naples, Italy; anna.annunziata@gmail.com (A.A.); pasquale.imitazione@gmail.com (P.I.); francesimioli@gmail.com (F.S.); giuseppefiorentino1@gmail.com (G.F.); 5 Department of Translational Medicine, University of Naples Federico II, 80138 Naples, Italy; laura.ferrentino95@gmail.com

**Keywords:** bronchiectasis, high-flow nasal therapy, bronchiectasis severity index

## Abstract

**Background/Objectives**: High-flow nasal therapy (HFNT) has been shown to reduce exacerbations of COPD and some evidence displays benefits in non-cystic fibrosis bronchiectasis (NCFB) patients. The present study aimed to compare the effectiveness of 12 months of home HFNT on the annual exacerbation rate between mild/moderate and severe NCFB patients, classified by the bronchiectasis severity index (BSI). Secondary outcomes were the evaluation of the dyspnea, pulmonary function, and sputum cultures in both groups. **Methods**: The study population included NCFB adult patients, with at least one severe exacerbation in the previous year on optimized therapy. NCFB exacerbations, dyspnea (mMRC score), pulmonary function test, and sputum cultures were assessed at baseline and after 12 months of HFNT. **Results**: A total of 86 NCFB patients were enrolled: 36 in the mild/moderate (BSI < 9) and 50 in the severe (BSI ≥ 9) group. A significant improvement in the annual exacerbation rate was found in both BSI ≥ 9 (*p* < 0.0001) and BSI < 9 cohorts (*p* < 0.0001), with a between-group difference of −1 (95% CI: −2 to 0) exacerbations per year (*p* = 0.0209). The change in the annual exacerbation rate was significantly correlated with BSI (*ρ* = −0.26, *p* = 0.0151) and with HFNT daily use (*ρ* = −0.22, *p* = 0.0460). The mMRC score significantly improved by −2 points (95% CI: −2 to −1) after treatment in both groups (*p* < 0.0001). The percentage of patients with *P. aeruginosa* colonization decreased from 34.9% to 27.9%. **Conclusions**: Long-term HFNT reduces the annual exacerbation rate in NCFB patients and its effectiveness increases alongside disease severity and daily use of HFNT.

## 1. Introduction

Non-cystic fibrosis bronchiectasis (NCFB) is a chronic respiratory disease characterized by the abnormal and permanent dilatation of the bronchi associated with persistent cough, sputum production, and recurrent infections [1]. The pathogenesis of bronchiectasis relies on a complex interaction between airway inflammation, infection, impaired mucociliary clearance, and lung damage [2]. NCFB prevalence alone or as a comorbid condition has increased by nearly 40% over the last decade, and it is associated with substantial increased morbidity, mortality, and economic burden [3,4,5].

While there are an increasing number of ongoing trials on antibiotics and anti-inflammatory drugs, improving mucociliary clearance still plays a crucial role in interrupting the bronchiectasis vicious vortex [6,7,8,9]. Strategies to facilitate the mobilization of secretions have been demonstrated to reduce the recurrence of exacerbations, critical events in the natural history of bronchiectasis associated with worse quality of life, more frequent hospitalizations, and increased mortality. High-flow nasal therapy (HFNT) is a noninvasive respiratory support that delivers heated and humidified gases, eventually blended with oxygen, at a high flow rate and has been shown to significantly improve mucociliary clearance [10]. More than a decade ago, Hasani and colleagues demonstrated, by a radio aerosol technique, a meaningful enhancement of tracheobronchial mucociliary clearance induced by three hours per day of HFNT for seven days [11]. Initially proposed as an alternative to standard oxygen therapy in the treatment of acute hypoxemic respiratory failure, HFNT has shown several important additional benefits including alveolar recruitment, reduction in dead space, carbon dioxide washout, increased mucus hydration, preserved ciliary function, and, at the same time, good patient comfort and tolerance [12,13,14,15,16,17,18,19]. Most studies have focused on COPD patients with hypoxemic respiratory failure with or without hypercapnia showing that HFNT, in addition to long-term oxygen therapy (LTOT), and in comparison to only LTOT, significantly reduces exacerbations and hospital admissions, improves dyspnea and health-related quality of life, and decreases hypercapnia [20,21,22]. In addition, in patients with COPD or bronchiectasis, HFNT, in comparison with the usual care, significantly lowers exacerbation frequency and ameliorates quality of life [23]. Emerging evidence demonstrates the long-term benefits of home HFNT for patients with bronchiectasis, including a reduction in exacerbations and hospital admission and an improvement in the quality of life [24,25,26,27]. However, the specific advantages of HFNT across different severities of bronchiectasis remain poorly understood and under-researched.

The aim of the present study was to evaluate the effectiveness of 12 months of domiciliary HFNT on the annual rate of exacerbations between mild/moderate and severe NCFB patients, classified according to the bronchiectasis severity index (BSI) [28]. Secondary outcomes of the study were the comparison between the two groups regarding the degree of dyspnea, functional parameters, and sputum microbiological cultures.

## 2. Materials and Methods

This retrospective observational study included patients with NCFB referred to two Italian outpatient clinics: the Policlinico “G. Rodolico-San Marco” University Hospital, Catania, and the Monaldi Hospital, Naples, utilizing data from the prospective ongoing databases of the two dedicated outpatients bronchiectasis facilities of the two centers. This study was performed in line with the principles of the Declaration of Helsinki. Adult patients (≥18 years) with a clinical history of chronic cough and sputum production, HRCT evidence of bronchiectasis, on treatment with optimized medical therapy and physiotherapy in accordance with ERS/ATS guidelines, and at least one severe exacerbation of BE (defined as an exacerbation requiring hospital admission) in the previous year, were enrolled [29].

Active smokers, patients with traction BE, cystic fibrosis, pulmonary diseases other than BE, chronic obstructive pulmonary disease (COPD) or bronchial asthma, unstable heart, kidney, or liver diseases and malignancies were excluded from the study.

Patients were assigned to two study cohorts according to the BSI [28], a prognostic index for morbidity and mortality, which considers the following nine variables: age, body mass index (BMI), forced expiratory volume in one second (FEV_1_) as % of predicted, previous hospital admission, previous exacerbations, dyspnea assessed by modified medical research council (mMRC) score, colonization with *Pseudomonas aeruginosa*, colonization with other organisms, and radiological severity (assessed by the number of involved lobes or by the presence of cystic bronchiectasis on chest CT). By summing the scores for each variable, patients are classified as mild (0–4 points), moderate (5–8 points), or severe (≥9 points) bronchiectasis. The study population was divided into two groups: mild/moderate (BSI <9 points) and severe (BSI ≥ 9 points).

All patients initiated HFNT using a dedicated device (myAirvo 2, Fisher&Paykel Healthcare, Auckland, New Zealand) and nasal cannula (Optiflow; Fisher&Paykel Healthcare, Auckland, New Zealand) with the size selected to occlude about 2/3 of the patient’s nostril. Flow rate and temperature, initially set at a flow rate of 45 L/min and a temperature of 37 °C, were modified according to patient comfort and tolerance. If necessary, oxygen supplementation was added and FiO_2_ adjusted to maintain SpO_2_ ≥ 92%. Patients were instructed to use the device for at least 6 h a day, preferably at night.

At the baseline visit, the following parameters were assessed: age, sex, BMI, smoking history, comorbidities (cardiovascular diseases and diabetes), BSI, aetiology of bronchiectasis, results of sputum cultures, concomitant medical treatment, and oxygen therapy.

At baseline and after 12 months of domiciliary HFNT, the following variables were evaluated: the number of exacerbations of bronchiectasis in the previous year, the degree of dyspnea, assessed by the mMRC score, functional parameters (FEV_1_% predicted), and sputum microbiological cultures.

### Statistical Analysis

Data are presented as mean ± standard deviation (SD) for normally distributed continuous variables and as median with interquartile range (IQR) for nonparametric continuous variables. Categorical variables are expressed as numbers (n) and percentages (%). The normality of the data was assessed using the Anderson–Darling test. Differences between treatment groups were evaluated using the unpaired Student’s *t*-test for normally distributed data and the Wilcoxon rank-sum or Mann–Whitney tests for nonparametric data. Fisher’s exact test was used to compare categorical variables. Median differences with 95% confidence intervals (95% CI) were calculated to assess treatment effects. Linear regression analysis was conducted to evaluate the association between BSI and the hours of HFNT use per day with changes in exacerbations, mMRC scores, and FEV_1_ (%). Spearman’s rank correlation coefficients (*ρ)* were calculated. No multicollinearity was found, as the variance inflation factor was ≤5 for all variables [30]. All statistical tests were two-tailed, and *p*-values < 0.05 were considered statistically significant. Statistical analyses and figures were generated using GraphPad Prism (version 10.1.0, GraphPad Software, San Diego, CA, USA) and SPSS (version 26, IBM Corporation, Armonk, NY, USA).

## 3. Results

### 3.1. Baseline Patient Characteristics and HFNT Settings

The study population included 86 patients with bronchiectasis, who were divided into two groups: 36 patients in the mild/moderate (BSI < 9 points) group and 50 in the severe (BSI ≥ 9 points) group. Patient baseline characteristics and comparisons between the BSI < 9 and BSI ≥ 9 groups are reported in Table 1.

The mean age was 67.5 ± 10.8 years, with patients in the BSI severe cohort being older than those in the mild/moderate group (70 ± 9.7 years vs. 63.8 ± 11.6 years, *p* = 0.0091). Overall, 59.3% of the patients were females. A history of smoking was more prevalent in the severe bronchiectasis group compared to the mild/moderate group (48% vs. 16.7%, *p* = 0.0030), as well as cardiovascular comorbidities being significantly more prevalent in the severe group (44% vs. 11.1%, *p* = 0.0017). The mean BSI for the entire population was 9.7 ± 4.3, with the score significantly higher in the severe group in comparison with the mild/moderate group (12.6 ± 2.7 vs. 5.4 ± 1.7, *p* < 0.0001). Regarding the aetiology, more patients with post-infective bronchiectasis were found in the BSI < 9 group (63.9% vs. 36%, *p* = 0.0158), and more patients with COPD were in the BSI ≥ 9 cohort (36% vs. 5.6%, *p* = 0.0008). Sputum cultures were positive for *Pseudomonas aeruginosa* in 48% of patients with severe bronchiectasis compared to 16.7% in the mild/moderate group (*p* = 0.0030). The intensity of inhaled treatment regimens, inhaled corticosteroids–long-acting beta-agonists (ICS–LABA) and/or long-acting muscarinic agonists (LAMA) or LABA/LAMA, differed significantly between the two groups, being prescribed in a significantly higher percentage of patients with severe bronchiectasis. The mean number of annual exacerbations was significantly higher in the severe group [4 (IQR: 2–5.3) vs. 2 (IQR: 1–4), *p* = 0.0003)], and 94% of the patients with BSI ≥ 9 were hospitalized in the previous year compared to none in the BSI < 9 group (*p* < 0.0001). Patients with severe bronchiectasis had worse FEV_1_% at baseline [57.5% (IQR: 44.3–74) vs. 71% (IQR: 58–84), *p*= 0.0018].

The HFNT settings and the hours of use per day across the groups are shown in Table 2. No differences in the flow rates, temperature, FiO_2_, and hours/day were detected between the mild/moderate and severe groups.

### 3.2. Long-Term Home HFNT Effectiveness According to Bronchiectasis Severity

**Figure 1:**
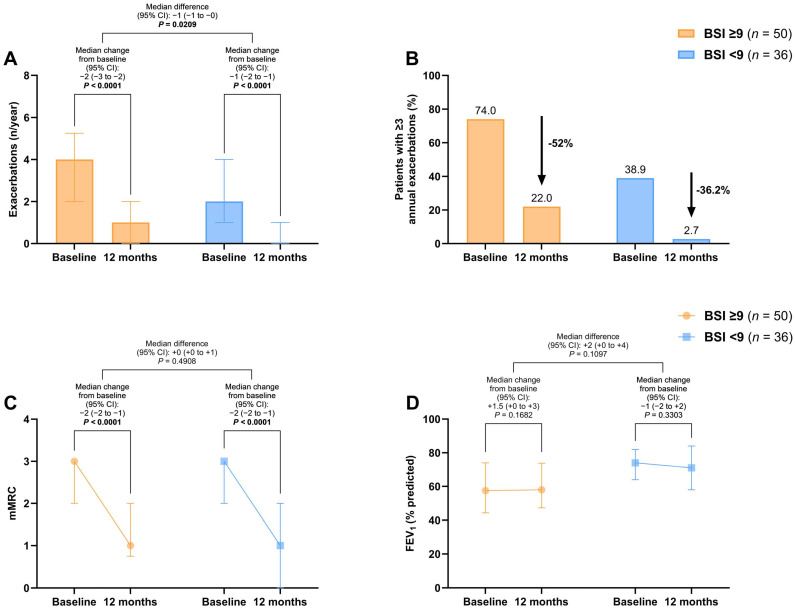
Changes in exacerbations (**A**,**B**), mMRC scores (**C**), and FEV_1_% (**D**) before and after 12 months of long-term home HFNT in patients with severe bronchiectasis (BSI ≥ 9) and mild/moderate bronchiectasis (BSI < 9). The proportion of patients with ≥3 annual exacerbations is presented as percentage of the total. All other variables are expressed as median and interquartile range. *p*-values in bold are statistically significant. Abbreviations: BSI, bronchiectasis severity index; mMRC, modified medical research council; FEV_1_, forced expiratory volume in the first second; HFNT, high-flow nasal therapy; CI, confidence intervals.

A significant improvement in the exacerbation rate at 12 months was found in both the BSI ≥9 [−2 (95% CI: −3 to −2), *p* < 0.0001] and the BSI <9 cohorts [−1 (95% CI: −2 to −1), *p* < 0.0001], with a between-group difference of −1 (95% CI: −2 to 0) exacerbations per year (*p* = 0.0209) (Figure 1A). The proportion of patients with ≥3 annual exacerbations decreased by 52% in the severe bronchiectasis group and 36.2% in the mild/moderate group (Figure 1B).

The mMRC dyspnea scale score significantly improved by −2 points (95% CI: −2 to −1) after treatment in both groups (*p* < 0.0001), with no significant difference between the severe and mild/moderate bronchiectasis patients (Figure 1C). No changes in FEV_1_% were observed before and after treatment (Figure 1D).

### 3.3. Correlations between Bronchiectasis Severity, Daily HFNT Usage, and Changes in Clinical Outcomes

**Figure 2:**
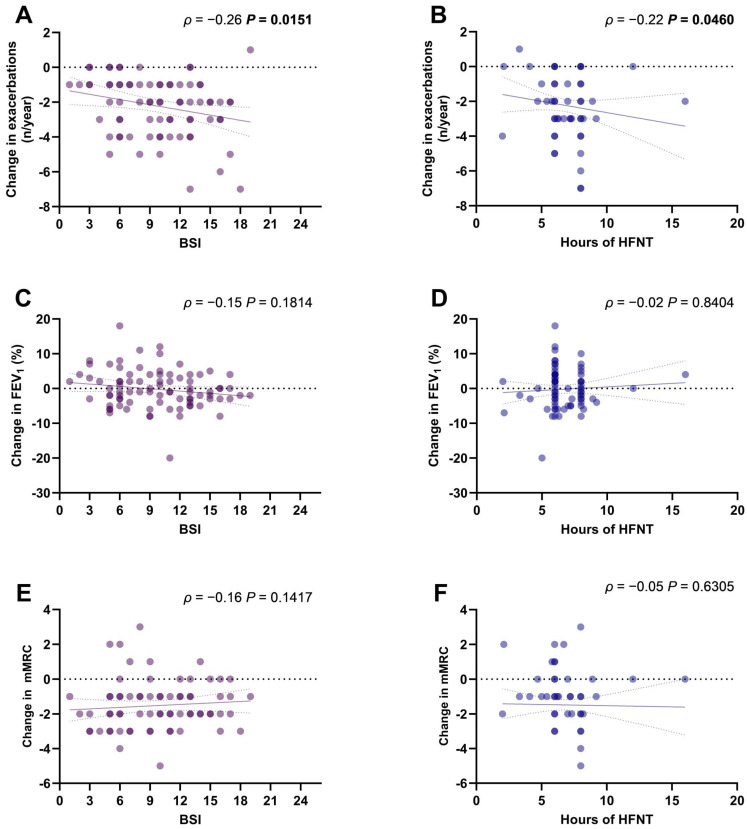
Scatter diagrams and regression lines (95% CI) of correlation between BSI and HFNT usage with changes in annual exacerbations rate (**A**,**B**), mMRC score (**C**,**D**), and FEV_1_% (**E**,**F**). *ρ*: Spearman coefficient. *p*-values in bold are statistically significant. Abbreviations: BSI, bronchiectasis severity index; mMRC, modified medical research council; FEV_1_, forced expiratory volume in the first second; HFNT, high-flow nasal therapy; CI, confidence intervals.

The change in the annual exacerbations rate was significantly correlated with the BSI (*ρ* = −0.26, *p* = 0.0151) (Figure 2A) and with the hours of HFNT use per day (*ρ* = −0.22, *p* = 0.0460) (Figure 2B). Changes in FEV_1_% (Figure 2C,D) and mMRC scores (Figure 2E,F) were not correlated with BSI or HFNT usage.

### 3.4. Sputum Microbiological Colonization before and after 12 Months of Long-Term Home HFNT

**Figure 3:**
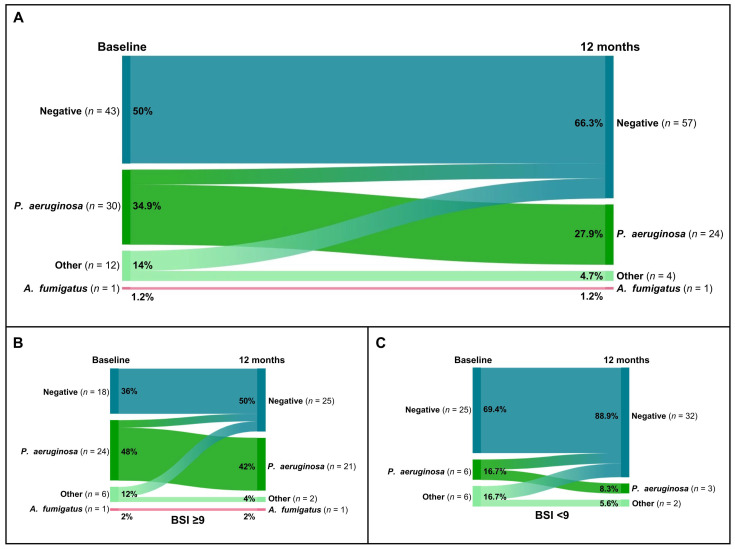
Sankey plots of microbiological colonization changes after 12 months of home HFNT in the overall cohort (**A**) and in the groups with severe bronchiectasis (BSI ≥ 9) (**B**) and mild/moderate bronchiectasis (BSI < 9) (**C**).

Overall, the percentage of patients with *P. aeruginosa* colonization decreased from 34.9% (30 of 86) to 27.9% (24 of 86) (Figure 3A). Additionally, among the 14% (12 of 86) of patients who were tested positive with other microorganisms, only 4.7% (4 of 86) tested positive for *P. aeruginosa* after 12 months of domiciliary HFNT (Figure 3A). Detailed changes in microbiological sputum status for patients with severe bronchiectasis and mild/moderate bronchiectasis are shown in Figure 3B and Figure 3C, respectively.

## 4. Discussion

The present study shows that long-term HFNT reduced the annual exacerbation rate in patients with mild/moderate and severe NCFB, classified accordingly to BSI, with its effectiveness increasing alongside the disease severity and the daily use of HFNT. Lowering the exacerbation frequency is considered one of the key goals in the management of NCFB, as exacerbations not only accelerate disease progression and lung function decline but also increase mortality and the economic burden on patients [31,32]. According to the literature, the 1-year mortality rate following a bronchiectasis exacerbation can be as high as 30% and this is significantly linked to the patient’s exacerbation rate and the number of hospitalizations in the previous year [33,34].

This study found that patients with a history of frequent exacerbations were more likely to benefit from HFNT. We demonstrated a greater reduction in exacerbation rate in the severe BSI patients, who had, at the baseline, a significantly higher number of exacerbations in the previous year compared to those with mild/moderate BSI patients. Additionally, we observed a relationship between the daily use of HFNT and the reduction in exacerbations, further supporting the preventive role of HFNT. The administration of heated and humidified gas with a high flow rate improves mucus hydration and ciliary function and can prevent mucus plugging, which can favor chronic bacterial infection. Of note, our study shows that the prolonged use of HFNT resulted in a reduction in the percentage of patients having positive sputum culture, particularly for *P. aeruginosa* at the 12-month time point. This result is very interesting, considering that colonization by *P*. *aeruginosa* is related to a worse prognosis and higher mortality [35,36]. Although the effects played by long-term domiciliary HFNT on some of the physio-pathological mechanisms can lead to airflow obstruction in bronchiectasis patients, the results of the present study did not show an improvement in the main spirometry parameter related to the airway caliber as FEV_1_, neither in the severe nor in the mild/moderate group. However, all patients enrolled in the study demonstrated an improvement in dyspnea, independent of the severity of the disease, confirming that HFNT improves breathing pattern, as previously reported [37,38]. It is important to outline that the severe bronchiectasis group included older patients, with a more frequent history of cigarette smoke exposure, and, probably as a result, with a more prevalent aetiology of bronchiectasis represented by COPD, a chronic airway disease in which the efficacy of HFNT in preventing exacerbations as well as hospital admissions, as has already been demonstrated [20,21,22,39]. These severe patients also had more cardiovascular comorbidities and more prevalent colonization by *P. aeruginosa*, thus representing a population at high risk of exacerbations, as shown by our data on their previous history of more frequent hospital admissions compared with the mild/moderate patients. Therefore, the efficacy of HFNT in this “fragile population” is significant, also considering the economic burden associated with the management of these patients, particularly in terms of antibiotics use, and unscheduled clinical visits and hospitalizations [5,40].

Of note, long-term HFNT was well tolerated by all patients enrolled in our study, and no adverse effects were reported, confirming previous evidence [38]. Most patients wanted to continue with HFNT after the 12-month trial period.

Our findings align with similar results reported in the post hoc analysis by Good and coworkers, analyzing the cohort of 45 patients with bronchiectasis treated with HFNT enrolled in the randomized controlled study conducted by Rea et al. [23,24]. The authors observed a significant reduction in the exacerbation rate (2.39 vs. 3.48 exacerbations/year, *p* = 0.034) and a notable improvement in quality of life (SGRQ −12.3 vs. −1.2, *p* = 0.028) with HFNT when compared to usual care [24].

Similar findings were documented in two other studies previously published by our study group [25,27]. Crimi et al. conducted a one-year observational, retrospective, case-controlled study involving 20 NCFB patients on long-term home HFNT and 20 controls on optimized medical treatment alone, matched by age, sex, BSI, and exacerbations in the previous year. The study demonstrated the effectiveness of domiciliary HFNT in reducing exacerbations and hospitalizations and showed a slight but statistically significant improvement in pulmonary function, compared to usual care [25]. Simioli et al., in their retrospective analysis of 78 patients with NCFB, demonstrated the effectiveness of two-year domiciliary treatment with HFNT in reducing the number of exacerbations. They observed a significant decrease in dyspnea according to the mMRC score without an improvement in pulmonary function parameters after two years [27]. A recent systematic review demonstrated that HFNT is more effective than usual care as a long-term strategy for reducing exacerbations and improving quality of life in patients with COPD and bronchiectasis, showing a good adherence profile and safety in the home settings [38].

The results of the present study represent additional important evidence on the effectiveness of long-term domiciliary HFNT in patients with NCFB, showing that its preventive effect on exacerbations increases with the disease severity, assessed by the BSI, and with the time of daily use, reinforcing the concept that patients with more severe bronchiectasis are those who benefit the most from HFNT treatment and that adherence is a key goal for improving treatment effects.

Our study has several limitations, mainly related to the relatively low sample size and the retrospective design that may limit the generalizability of the results. Moreover, the single-arm study design does not allow us to discriminate the effects of HFNT from that of the concomitant pharmacological treatment, which was significantly higher, as expected, in severe patients compared with mild/moderate patients. Furthermore, patients with mild/moderate BSI were lower compared to those with severe BSI and we cannot exclude that this slight difference might affect the results. Lastly, it is possible that certain long-term benefits or side effects would not have been observable during the 12-month follow-up. However, we conducted a real-world assessment of the effectiveness of HFNT in patients with different severity of NCFB, including those with multiple comorbidities. Additional prospective studies with a larger sample size are required to define the role of HFNT further compared to usual care in this cohort of patients. Finally, longer study designed to evaluate other important outcomes as the impact of HFNT on the mortality rates of bronchiectasis patients or on the cardiovascular event risk are needed, as well as to define the better timing for HFNT initiation following an exacerbation of bronchiectasis.

## 5. Conclusions

Long-term HFNT significantly reduces the annual exacerbations rate in patients affected by bronchiectasis, with its effectiveness increasing with the severity of the disease and the time use of HFNT.

## Figures and Tables

**Table 1:** Baseline clinical characteristics.

	All (*n* = 86)	BSI ≥ 9 (*n* = 50)	BSI < 9 (*n* = 36)	*p*-Value *
**General characteristics**				
Age, years, mean (SD)	67.5 (10.8)	70 (9.7)	63.8 (11.6)	**0.0091**
Female, *n* (%)	51 (59.3)	31 (62)	20 (55.6)	0.6573
BMI, kg/m^2^, mean (SD)	24.6 (4.4)	24.5 (4.6)	24.8 (4.3)	0.7642
Smoking history, *n* (%)	30 (34.9)	24 (48)	6 (16.7)	**0.0030**
**Comorbidities**				
Cardiovascular disease, *n* (%)	26 (30.2)	22 (44)	4 (11.1)	**0.0017**
Diabetes, *n* (%)	15 (17.4)	11 (22)	4 (11.1)	0.2537
**Long-term oxygen therapy, *n* (%)**	9 (10.5)	8 (16)	1 (2.8)	0.0732
**Bronchiectasis assessment**				
BSI, mean (SD)	9.7 (4.3)	12.6 (2.7)	5.4 (1.7)	**<0.0001**
**Bronchiectasis aetiology, *n* (%)**				
Idiopathic	13 (15.1)	8 (16)	5 (13.9)	0.9999
Post-infective	41 (47.7)	18 (36)	23 (63.9)	**0.0158**
COPD	20 (23.3)	18 (36)	2 (5.6)	**0.0008**
Immunodeficiency	2 (2.3)	2 (4)	0 (0)	0.5075
Primary ciliary dyskinesia	7 (8.1)	6 (12)	1 (2.8)	0.2307
Inflammatory bowel disease	1 (1.2)	1 (2)	0 (0)	0.9999
Severe asthma	2 (2.3)	1 (2)	1 (2.8)	0.9999
**Sputum cultures, *n* (%)**				
Negative	43 (50)	18 (36)	25 (69.4)	**0.0042**
*Pseudomonas aeruginosa*	30 (34.9)	24 (48)	6 (16.7)	**0.0030**
Other bacteria	12 (14)	6 (12)	6 (16.7)	0.3567
*Aspergillus fumigatus*	1 (1.2)	1 (2)	0 (0)	0.9999
**Inhaled Therapies**				
ICS–LABA, n (%)	25 (29)	11 (22)	14 (38.9)	0.0990
ICS–LABA and/or LAMA, *n* (%)	32 (37.2)	24 (48)	8 (22)	**0.0231**
LAMA + LABA, *n* (%)	6 (7)	6 (12)	0 (0)	**0.0379**
**Clinical outcomes**				
Annual exacerbations, median (IQR)	3 (1–5)	4 (2–5.3)	2 (1–4)	**0.0003**
Annual hospitalizations, *n* (%)	47 (54.7)	47 (94)	0 (0)	**<0.0001**
mMRC dyspnea scale, median (IQR)	3 (2–3)	3 (2–3)	3 (2–3)	0.5672
FEV_1_, % predicted, median (IQR)	68.5 (50.3–77.8)	57.5 (44.3–74)	71 (58–84)	**0.0018**

* For comparisons between BSI ≥ 9 and BSI < 9 groups. *p*-values in bold are statistically significant. Abbreviations: BMI, body mass index; COPD, chronic obstructive pulmonary disease; BSI, bronchiectasis severity index; ICS, inhaled corticosteroids; LABA, long-acting beta-agonist; LAMA, long-acting muscarinic antagonist; mMRC, modified medical research council; FEV_1_, forced expiratory volume in the first second; SD, standard deviation; IQR, interquartile range.

**Table 2:** HFNT settings.

	All (*n* = 86)	BSI ≥ 9 (*n* = 50)	BSI < 9 (*n* = 36)	*p*-Value *
High-flow rate, L/min, median (IQR)	45 (45–50)	45 (45–45)	45 (45–50)	0.3414
Temperature, °C, median (IQR)	34 (34–37)	34 (34–37)	34 (34–37)	0.1957
FiO_2_, %, median (IQR)	21 (21–21)	21 (21–21)	21 (21–21)	0.4381
Hours/day, median (IQR)	6 (6–8)	6 (6–8)	6 (6–8)	0.5877

* For comparisons between BSI ≥ 9 and BSI < 9 groups. Abbreviations: BSI, bronchiectasis severity index; FiO_2_, fraction of inspired oxygen; IQR, interquartile range.

## Data Availability

Data are available on request due to privacy/ethical restrictions.
